# Barriers and bridges for sustaining functional habitat networks: A macroecological system analysis of wet grassland landscapes

**DOI:** 10.1002/ece3.8801

**Published:** 2022-04-06

**Authors:** Per Angelstam, Michael Manton, Ingrid Stjernquist, Tómas Grétar Gunnarsson, Richard Ottvall, Mats Rosenberg, Ole Thorup, Per Wedholm, Jaanus Elts, Davis Gruberts

**Affiliations:** ^1^ School for Forest Management Swedish University of Agricultural Sciences Skinnskatteberg Sweden; ^2^ Department of Forestry and Wildlife Management Inland Norway University of Applied Sciences Koppang Norway; ^3^ 105468 Faculty of Forest Science and Ecology Vytautas Magnus University Akademija Lithuania; ^4^ 7675 Environmental and Resource Dynamics Group Department of Physical Geography Stockholm University Stockholm Sweden; ^5^ South Iceland Research Centre University of Iceland Laugarvatn Iceland; ^6^ Private Firm Höör Sweden; ^7^ Rosenberg Natur Vintrosa Sweden; ^8^ Amphi Consult Ribe Denmark; ^9^ Örebro Municipality Örebro Sweden; ^10^ Birdlife Estonia Tartu Estonia; ^11^ 37546 Department of Zoology Institute of Ecology and Earth Sciences University of Tartu Tartu Estonia; ^12^ Department of Chemistry and Geography University of Daugavpils Daugavpils Latvia

**Keywords:** biodiversity, conservation, governance, landscape restoration, macroecology, meta‐conspiracy, power, social–ecological system

## Abstract

This study aims at supporting the maintenance of representative functional habitat networks as green infrastructure for biodiversity conservation through transdisciplinary macroecological analyses of wet grassland landscapes and their stewardship systems. We chose ten north European wet grassland case study landscapes from Iceland and the Netherlands in the west to Lithuania and Belarus in the east. We combine expert experiences for 20–30 years, comparative studies made 2011–2017, and longitudinal analyses spanning >70 years. Wader, or shorebird, (*Charadrii*) assemblages were chosen as a focal species group. We used evidence‐based knowledge and practical experience generated in three steps. (1) Experts from 8 wet grassland landscapes in northern Europe's west and east mapped factors linked to patterns and processes, and management and governance, in social‐ecological systems that affect states and trends of wet grasslands as green infrastructures for wader birds. (2) To understand wader conservation problems and their dynamic in wet grassland landscapes, and to identify key issues for successful conservation, we applied group modeling using causal loop diagram mapping. (3) Validation was made using the historic development in two additional wet grassland landscapes. Wader conservation was dependent on ten dynamically interacting ecological and social system factors as leverage points for management. Re‐wetting and grazing were common drivers for the ecological and social system, and long‐term economic support for securing farmers’ interest in wader bird conservation. Financial public incentives at higher levels of governance of wetland management are needed to stimulate private income loops. Systems analysis based on contrasting landscape case studies in space and over time can support (1) understanding of complex interactions in social‐ecological systems, (2) collaborative learning in individual wet grassland landscapes, and (3) formulation of priorities for conservation, management, and restoration.

## Graphical abstract

We used evidence‐based knowledge and practical experience generated in three steps. (1) Experts from 8 wet grassland landscapes mapped factors that affect states and trends of wet grasslands as green infrastructures for wader birds. (2) We then applied group modelling using causal loop diagram mapping. (3) Validation was made using the historic development in two additional wet grassland landscapes.

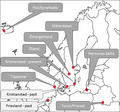

## 1 INTRODUCTION

Research into broad‐scale general patterns and trends of ecological systems, the processes that underlie them, and relationships with governance, planning, and management in social systems, has long called for new modes of knowledge production and learning (Brown, 1995; Gibbons et al., 1994). Systems analyses (Maani & Cavana, 2000) defining both shallow and deep leverage points for sustaining ecological systems is an effective approach (Meadows, 2008). Ultimately, a resilient ecosphere is a foundation for landscapes’ social and economic systems (United Nations, 2015). Maintaining the integrity of intact ecosystems with representative and functional habitat networks supporting biodiversity conservation and human well‐being is thus a key component of proactive global and national environmental strategies (Watson et al., 2018). Policies and terms like ecological networks and greenways (Jongman et al., 2004, 2011), ecological infrastructure (Angelstam, Barnes, et al., 2017), and green infrastructure (European Commission, 2013) capture this key component. Nevertheless, a traditional focus on protected areas and species and not functional habitat networks prevails (Harvey et al., 2017). Thus, habitat alteration, fragmentation, and loss continue, which results in reduced functionality of habitat networks (Angelstam & Manton, 2021; Beyer et al., 2020; Correa Ayram et al., 2016).

According to Grint (2008), decision‐makers have three types of problems: tame, critical, and wicked. While for the first two, being less or more urgent to handle, there are common well‐established best practices that can be scaled up. In contrast, for wicked problems, there is no consensus of the problem, and there is disagreement among actors and stakeholders, including researchers with different lenses (Maxwell et al., 2020). Nikolakis and Innes (2020:13) listed three components for tackling wicked problems: collaborative governance, adaptive leadership, and holistic system‐based thinking. Comparative studies of multiple landscapes as social‐ecological systems are effective for holistic systems analyses about the green infrastructures (Angelstam et al., 2019, 2021; Dawson et al., 2017, 2021).

Already Von Thünen (1910) observed that the types and intensities of land use were related to the distance from the market. The European continent is a prime example of a region with a diverse history of land use and management. Loss of habitats in Europe is related to a generally expanding human footprint in terms of increasingly intensified land use from the core to the periphery of economic development (Bobiec et al., 2019; Gunst, 1989; IPBES, 2019). This takes place in spite of considerable resources being spent on nature conservation through the creation of protected areas (Kati et al., 2015), “re‐wilding” (Perino et al., 2019), and habitat/landscape restoration (Emanuelsson, 2009) with the aim to maintain functional habitat networks. Europe thus has distinct gradients of alteration, fragmentation, and loss of remnants of both traditionally multifunctional cultural landscapes and naturally dynamic forest landscapes (Angelstam, Khaulyak, et al., 2017; Angelstam, Naumov, et al., 2018, Angelstam et al., 2021; Edman et al., 2011; Manton et al., 2019; Manton & Angelstam, 2021; Puumalainen et al., 2003). Intensification of land management in European centres of economic growth is thus responsible for declines and even local extinction of species (Storkey et al., 2012; Thorup, 2006) and modification of trophic interactions (Angelstam, Manton, et al., 2017; Manton et al., 2019). In comparison, land management and use in European peripheries have developed slower, are generally less intensive, and have better retained ecological patterns and processes in landscapes (Angelstam et al., 2021; McDonnell et al., 2008; Valasiuk et al., 2018).

Transformation of naturally dynamic ecosystems into anthropogenic land covers has both negative and positive consequences for ecosystem processes, habitats, and species in Europe (Angelstam, Naumov, et al., 2018; Price, 2003). Transitioning natural forest ecosystems by management to produce industrial raw material generally leads to loss of biodiversity (Naumov et al., 2018). In contrast, the emergence of agriculture based on animal husbandry often created cultural landscapes with large areas of high conservation value farming areas, such as wet grasslands and other naturally open landscapes with grasslands and heaths used for haymaking and as pastures for grazing (Emanuelsson, 2009; Eriksson & Cousins, 2014; Hejcman et al., 2013; Manton & Angelstam, 2018). To benefit from the high nutrient content of soils, wet grasslands were thus expanding in river deltas, along seashores, in flooded areas along rivers and streams, or through man‐made seepage areas (Emanuelsson & Möller, 1990). However, driven by the agricultural and industrial revolutions over the last century, large differences among European agricultural landscapes have developed. Thus, countries outside the EU, like some former parts of the USSR in the eastern periphery of Europe have retained traditional practices (Valasiuk et al., 2018). Iceland in the northern periphery has developed its own agricultural policies and even encouraged the expansion of animal husbandry through expanded grassland areas (Fridriksson, 1972; Helgadóttir et al., 2013). This has resulted in considerable variation in the state and trends of wet grassland vegetation patterns, such as patch size and spatial configuration, and processes, such as re‐wetting, grazing, mowing, and predation in cultural landscapes in Europe (Manton & Angelstam, 2018, 2021; Manton et al., 2016, 2019; Smart et al., 2006), and the associated population trends of waders, or shorebirds (*Charadrii*) (Thorup, 2006; Verkuil, Karlionova, et al., 2012; Verkuil, Piersma, et al., 2012).

Noting the limited success of traditional conservation management based on protected areas and species only, Harvey et al. (2017) stressed the need for integrative approaches focusing on ecological networks as a conservation target. In particular, this would allow for better conceptual bridging of ecosystem‐level supporting processes and emerging services. With annual global scale migration routes spanning multiple continents (Verkuil, Karlionova, et al., 2012; Verkuil, Piersma, et al., 2012; van Vliet et al., 2015), waders are excellent model organisms, often used as indicators of ecosystem health (Sutherland et al., 2012). To address the threats faced by deteriorating semi‐natural grasslands in breeding areas, knowledge production and learning should also pay more attention to the inherent social‐ecological complexity of them (Herzon et al., 2021). The dynamic cultural wet grasslands were well suited for wader birds, at different points in time depending on the land use, due to a range of factors like abundant food, favorable hydrological regimes, grass mowing for fodder, grazing, and livestock churning the soil (Emanuelsson, 2009; Laidlaw et al., 2015; Leito et al., 2014). Regional differences in the timing of cultural wet grassland expansion and decline have thus led to frontiers of emergence and degradation of wet grassland landscapes, and very few wader populations have remained viable or shown increases (Gunnarsson et al., 2005; Johannesdottir et al., 2019). The sequence of rise and fall of grasslands is paralleled by the pattern that some European regions exhibit declines in breeding migratory waders (Gill et al., 2007; Manton & Angelstam, 2021; Schekkerman et al., 2009). Waders have an umbrella species function: management for threatened waders has a strong supporting impact on meadow plants and amphibians (Rannap et al., 2017). In Europe, the ruff (*Philomacus pugnax*) and black‐tailed godwit (*Limosa limosa*) function as flagship species (Schlagloth et al., 2018) for wader bird communities in wet grasslands (Van der Vliet, 2015). Optimal breeding habitat for ruff and black‐tailed godwit is thus often a suitable breeding habitat for other waders, such as dunlin (*Calidris alpina*), redshank (*Tringa totanus*), and lapwing (*Vanellus vanellus*).

The aim of this study is to analyze the dynamics of multiple wader landscapes as social‐ecological systems that determine the distribution and abundance of wader populations that depend on wet grasslands as a functional green infrastructure. Using a transdisciplinary approach, researchers and practitioners collaborated to understand how different drivers of wader bird distribution and abundance are interlinked. We integrate macroecological methods, comparative analyses by experts, meta‐analyses, peer‐review publications, combined with a multiple landscape case study approach based on reviewing the knowledge from a suite of wet grassland landscapes at different stages of development of green infrastructure functionality, system analysis using causal loop modeling, and validation using landscape history reviews. The discussion focuses on the need to address the complexity of wet grasslands as social‐ecological systems for biodiversity conservation and human well‐being, and how systems analysis can contribute.

## 2 METHODOLOGY

### 2.1 Multiple landscapes as case studies

Research aimed at studying relationships among different variables, which explain an outcome variable, should be based on data collection representing contexts with sufficient variation of parameter values in the variables of interest (Yin, 2014). However, the design of dose‐response studies can determine the conclusions (Angelstam, Pedersen, et al., 2018). Studies of factors affecting habitat network functionality therefore require study areas that mirror both sufficient spatial extents and different levels of land‐use intensification. This calls for a macroecological approach (Brown, 1995), which relies on multiple landscapes as case studies in the regional gradient of landscape history (Manton et al., 2019). Therefore, the regional diversity of landscapes and regions on the European continent provides unique opportunities to develop evidence‐based knowledge for biodiversity conservation. Noting the issue that social‐ecological research is composed mainly of consolidated groups of scientists from developed countries leading work of peripheral barely consolidated groups (Santiz et al., 2021) this study includes a geographically and thematically broad portfolio of co‐authors.

**FIGURE 1:**
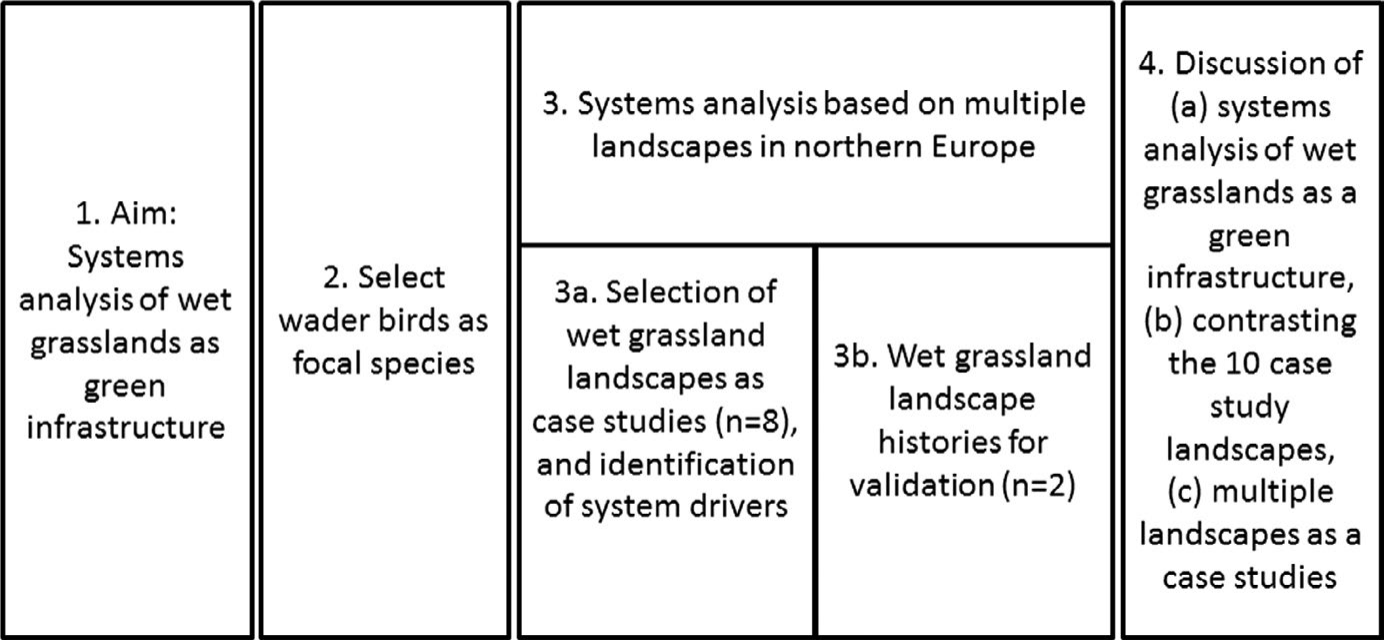
Methodological approach

**FIGURE 2:**
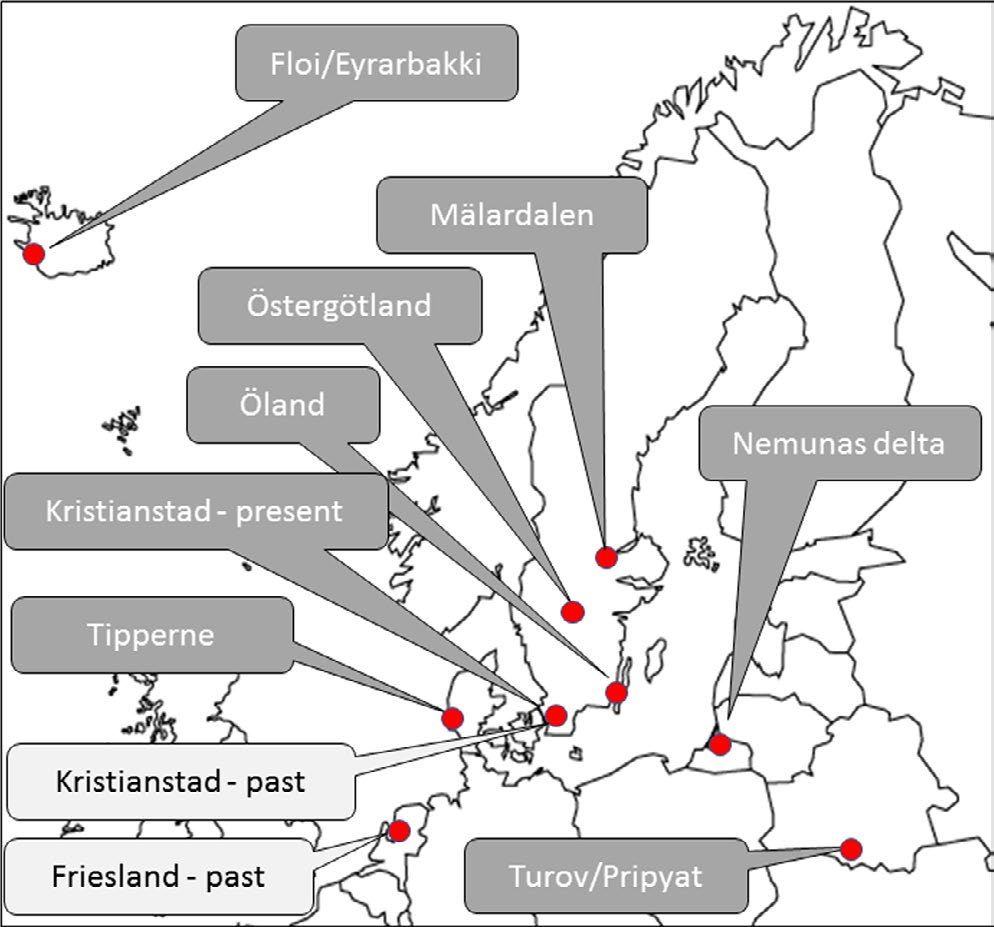
Wet grassland case study landscapes in northern Europe. Dark gray boxes with white text represent contemporary case studies used for causal loop modeling with both researchers and practitioners. Light gray boxes with black text represent wet grassland landscapes of the past are used for validation

Following the terminology of Stake (2003), each landscape unit of study is a “bounded” separate entity in terms of place and space with physical boundaries hosting a neighborhood, and planning and management organizations, or histories. With a single case study approach, one can do in‐depth exploration of a specific bounded system. Based on several different cases as a “collective case design” (Figures 1 and 2), with several instrumental bounded cases, we aimed at gaining in‐depth understanding of the opportunities and barriers for GI maintenance; much more than any single case can provide (Chmiliar, 2010; Yin, 2014).

**FIGURE 3:**
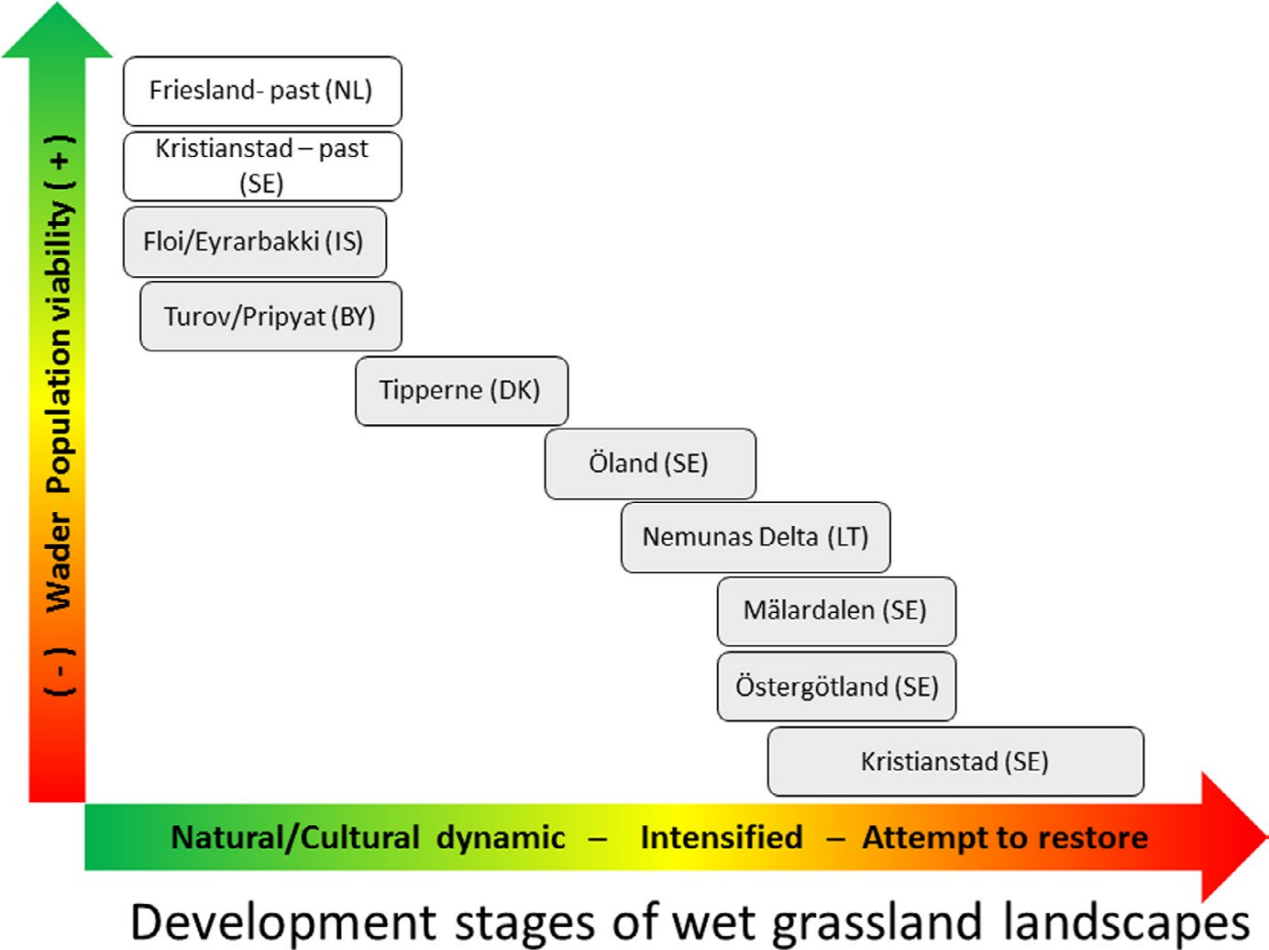
Illustration of the development of wet grassland case study landscapes (Figure 2, see also Manton & Angelstam, 2021) from natural via anthropogenic to degraded, followed by attempts towards restoration

Focusing on the variation among contemporary landscapes in northern Europe's west and east we selected 8 case study areas (Appendix S1), which represent temporal changes in wet grasslands and wader population trajectories over the past 20–30 years. Additionally, the status of two wet grassland landscapes 70–200 years ago was used for validation (Appendix S2); (see Figure 2). The 8 wet grassland landscapes represent different stages of development and degradation and are subject to different governance and management efforts towards wet grassland conservation and restoration. To illustrate the relative level of transition from functional habitat networks to degraded wet grassland systems, we compiled information about trends in the amount of habitat, the level of habitat fragmentation using the presence of large (>1 km^2^) wet grassland patches (Rannap et al., 2017), and the presence and trends of different wader species (see Manton & Angelstam, 2021) and the proportion of the regional species pool. Based on this we created a ladder of predicted relative wader population sustainability (Figure 3). Using the idea of Europe as a “time machine” and a laboratory for learning (Angelstam et al., 2011), the choice of case study landscapes thus followed the recommendation of information‐oriented selection with critical cases (Flyvbjerg, 2006). This approach maximizes information from a small number of cases, and these are selected due to their information content and for their generalization characteristics.

### 2.2 Mapping of drivers, systems analysis, and validation

To support a systems perspective on landscapes as coupled human and nature systems, we chose the multi‐tiered social‐ecological system (SES) framework (Partelow, 2018). This is useful for diagnosing both social systems focusing on governance interactions at multiple levels and outcomes in ecological systems with a focus on their sustainability at multiple scales. The SES framework has evolved into a systematic approach to understand how different SESs can be sustained (McGinnis & Ostrom, 2014). We used a multi‐method approach in three steps to understand the dynamics of wader populations in different social‐ecological contexts.

The first step was to gather researchers and practitioners working within the 8 selected wet grassland landscapes for a 3‐day workshop in December 2016 to synthesize expert knowledge (Pearce‐Higgins et al., 2017). We collectively listed variables capturing drivers in social‐ecological systems affecting waders in local wet grassland landscapes. The workshop participants were interviewed using a semi‐structured method (Flick, 2006) focusing on drivers which determine the distribution and abundance of breeding waders and the grassland dynamics on the local site, and effects of management and government initiatives. The results from the interviews to extract experts’ experience were complemented with supporting evidence from the literature (Appendix S1). As a framework to list variables as drivers in ecological systems we divided them into pattern and process (Turner, 1989) at local, landscape and regional scales. For social system drivers, we focused on governance and management at different levels.

Second, we used system dynamics and a group modeling approach (Hovman, 2014; Maani & Cavana, 2000; Sterman, 2000) collaboratively involving both nonacademic experts and researchers during the same workshop to develop conceptual models covering social, economic, and ecological aspects of wet grassland governance and management aimed at maintenance of a functional green infrastructure for wader bird populations. Applying a systems dynamics approach, the structures and nonlinearity of wet grasslands as complex socio‐ecological systems can be analyzed and the cause‐relationship, feedback loops, and leverage points identified. The group modeling method allows all the participants to jointly define the system and its boundaries (Reed, 2008). By using Causal Loop Diagrams (CLDs), the complex dynamic research question can be structured and simplified (Haraldsson & Sverdrup, 2004). This process means that the participants are directly involved in the modeling work and jointly develop and review their understanding of the wet grassland/wader system, and the drivers and feedback. In the causal loop diagrams, each causal link has a polarity (+) when the direction of effect of the dependent variable is the same as the independent variable, and (−) when the direction is the opposite. The polarity of each feedback loop is essential for understanding the system's behavioral directionality, resulting in the magnification of the original effect (a reinforcing loop) or an equilibrating response (a balancing loop) (Sterman, 2000). Using group modeling allows the individual insights to expand outside their experience fields, thus developing a joint systems‐based understanding of the complex problem (Elbakidze et al., 2015; Rouwette et al., 2011).

At the start, the participants formulated the problem articulation through three social‐ecological questions for the modeling sessions, (1) What determines the breeding success of waders? (2) How can optimal land management for waders be achieved? (3) How to generate community interest in wader conservation? The facilitator drew and adjusted the CLDs on a whiteboard following the participants’ discussion about cause and effect, and critique and improvement. After the workshop, the CLDs were sent out to the participants for a final evaluation. This method, with continuous and collective peer‐review, allows the understanding of the system under study by all participants, and to be presented unambiguously and transparently.

The modeling was guided by three assumptions. The first is that evidence‐based knowledge about flagship waders such as the ruff and the black‐tailed godwit (Thorup, 2006; Verkuil, Karlionova, et al., 2012; Verkuil, Piersma, et al., 2012) can be used to identify factors needed to sustain viable populations of waders on wet grasslands in general during the breeding season. The second assumption is that breeding success, and not adult survival during the nonbreeding season, is the key driving factor (Roodbergen et al., 2008). The third assumption is that no radical changes in pressures and threats at the wader winter area have been identified during the past 2–3 decades, which is the period that this study represents.

Third, we validated the results from the comparative analysis of eight case study landscapes through a comparison with the long‐term history of two additional wet grassland landscapes (see Appendix S2).

## 3 RESULTS

### 3.1 Key factors affecting wader bird populations

The group discussions on the case studies clearly showed that experts’ experience was that wader bird population dynamics are dependent on both ecological and social system factors. The interview step identified ten key social‐ecological system variables (or drivers) for sustainable wader bird breeding in the 8 case study landscapes (Table 1). Ecological factors included soil quality, habitat fragmentation, predation pressure, size of wet grassland, edge effects, water condition factors (water table), and social system drivers (human management through grazing, mowing, draining). The interview results clearly showed the interconnection among drivers, and the complexity of the social‐ecological system, which was further analyzed during the group modeling.

**TABLE 1:** Presence (1) and absence (0) of ecological and social system variables as drivers identified for wader population dynamics sorted from local (top) to regional (bottom) across eight current case study areas

System	Variables	Iceland, Floi	Belarus, Pripet	Denmark Tipperne	Sweden, Öland	Lithuania, Nemunas delta	Sweden, Mälardalen	Sweden, Östergötland	Sweden, Kristianstad
Ecological	*Soil quality*	*1*	*1*	0	*0*	*1*	*1*	*1*	*1*
	*Water table*	*1*	0	0	*1*	1	*1*	*1*	1
	*Wet area size*	0	1	*1*	1	1	1	1	1
	*Grazing*	*1*	*1*	*1*	*1*	*1*	*1*	*1*	*1*
	Vegetation height	0	0	1	0	0	0	1	1
	Concealment of eggs	0	0	1	1	0	0	0	0
	Patch diversity/mosaic	1	0	0	0	0	0	0	1
	*Predators (abundance)*	*1*	*1*	*1*	*1*	*1*	*1*	*1*	*1*
	*Predator species (numbers)*	*0*	*1*	*1*	*1*	*1*	*1*	*1*	*1*
	*Nest predation*	*1*	*1*	*1*	*1*	*1*	*1*	*1*	*1*
	*Edge effects*	*0*	*0*	*1*	*0*	*0*	*1*	*1*	*1*
	*Habitat fragmentation*	*1*	*1*	*0*	*1*	*1*	*1*	*1*	*1*
	Climate	1	0	1	0	0	0	0	0
Social	*Draining*	*0*	*1*	*0*	*1*	*1*	*1*	*1*	*1*
	*Mowing*	*0*	*0*	*1*	*1*	*1*	*1*	*1*	*1*
	Fertilizing	1	0	0	0	0	1	1	1
	Agriculture	1	1	0	1	1	1	1	1
	*Human management*	*1*	*1*	*1*	*0*	*1*	*1*	*1*	*1*
	Predator nest boxes	0	0	1	0	0	1	0	1
	Predator control	0	0	1	1	0	0	0	1
	Land owner interest	0	0	0	1	0	1	1	1
	Recreational use	0	0	0	1	0	1	1	1
	Restoring wetlands	0	0	0	1	1	0	0	1
	Agroenvironm. subsidies	0	0	1	1	0	1	1	1

Note

The variables marked in italic were used as a basis in the first of several group modeling sessions.

### 3.2 The ecological system

#### 3.2.1 Pattern

##### Habitat patch quality

The key qualities were water table, plant community composition, and structure and soil quality (Table 1). Availability of water and appropriate vegetation structure on different scales were described as key factors determining patch quality. This is in line with Leito et al. (2014) showing that wader abundance was most strongly related to water level as a key factor for breeding habitat quality both positively and negatively. For some wader species, shallow open ponds were important for breeding success. Ivask et al. (2007) found that the water table, and flooding events, regulates resource availability for waders. The wader food resources are also dependent on overall soil quality, or nutrient‐rich patches in the landscape. Soil quality regulates patch vegetation composition, structure, and plant growth capacity. Berg and Hiron (2012) identified a dynamic relationship between water table, soil quality, and hydrological regime controlling plant community composition and structure.

##### Patch diversity

Waders use several different vegetation types to fulfill their needs during breeding. A diverse landscape structure including different patch types is therefore a key driver for breeding success (Laidlaw et al., 2015). For example, while the habitat needs to be wet and open, a protective vegetation structure for the chicks is essential during the breeding period and nests of some species require concealment. A set of wet patches around optimal feeding patches may reduce predation as wet barriers tend to lower the searching efficiency of those predators using mainly smell as a cue, e.g., red fox (*Vulpes vulpes)*.

##### Fragmentation and patch size

Large (≥100 ha) and wide (mean width ≥200 m) meadows were an overall positive factor for favorable wader breeding conditions (see also Manton & Angelstam, 2018; Rannap et al., 2017). An open area without woody vegetation providing perches decreases predation risk. Due to changes in agricultural techniques and intensity over the last two centuries, the fragmentation of wet grasslands has increased. For example, Manton and Angelstam (2018) used historical maps and agricultural statistics to show how the wet grassland in the Kristianstad case has been continuously lost and fragmented. Combining fragmentation and patch size they estimated a 98% decline in semi‐natural meadow habitat network functionality during the last two centuries. Emanuelsson (2009) showed how European cultural wetland landscapes have continuously been fragmented due to agricultural development and the decreased need for hay from natural meadows.

#### 3.2.2 Process

##### Water table fluctuation

This driver is closely linked to the habitat quality factor. Flooding increases the meadow nutrient condition, which improves hay production (Emanuelsson, 2009; Manton & Angelstam, 2018). The timing of the water table fluctuation over the breeding season is important for wader breeding (Eglington et al., 2008). Too deep water in spring forces the returning waders to seek other breeding areas. Drained wetlands lack this variation. Widespread drainage of wetlands for intensive agriculture has led to a drastic decline in the area of species‐rich wet grassland and transformed species compositions (Manton et al., 2021).

##### Grazing

Grazing by livestock is a process to keep vegetation height at an optimal level over the breeding season, both for wader food supply and nest concealment. Historically, wet grasslands’ provision of pastures and meadows was enhanced to satisfy the needs for animal husbandry for food and manure production. Wet grasslands were thus managed by grazing and mowing (see below). In the absence of grazing, shrubs and tall grass species encroach, and habitat suitability is reduced (Leito et al., 2014). Experiences from the Mälardalen case study (Berg et al., 2002) indicate that the timing of grazing in spring affect the breeding success of wader populations. However, both livestock used for grazing and overstocking may have a potential negative effect. For example, studies from Iceland show that sheep may eat wader eggs and horses can trample the nests when grazing (Katrínardóttir et al., 2015) and a study in Finland showed that trampling of dunlin nests had a significant negative effect on hatching success (Pakanen, 2011; Pakanen et al., 2011).

##### Predation

The factor of predation can be divided into predator abundance, the number of predator species, and the amount of nest predation (Table 1). A high predator species diversity with different food preferences opens up for predation on adults, and chicks and nest predation. Predation on adults indirectly affects the chick population. Depending on the case study area, predator species are corvid birds, foxes (*Vulpes vulpes*, *V*. *lagopus*), skuas (*Stercorius* spp.), peregrine falcon (*Falco peregrinus*), harriers (*Circus* spp.), gulls (*Larus* spp.). Corvids are identified as an indicator species for nest and chick predation (Manton & Angelstam, 2021; Manton et al., 2019) while peregrine falcon can be used as a predation indicator for adult waders (Manton et al., 2016; Svahn, 2016). The abundance of corvid birds varied considerably among Northern European landscapes due to the availability of anthropogenic food (Andrén, 1992; Atkinson et al., 2005; Manton et al., 2019; Marzluff & Neatherlin, 2006). The variation in corvid abundance is also reflected in rates of artificial nest predation (Manton & Angelstam, 2021).

##### Climate change

Smart and Gill (2003) listed a range of potential impacts of climate change on breeding waders. In this study change in winter temperature due to climate change was identified as a negative driver in the Danish case in terms of increased tree growth and therefore higher predation rates and decreased visibility. In Iceland, rapid shrub encroachment, which negatively affects waders, is probably both due to warmer temperatures and reduced sheep grazing (Gunnarsson, 2020).

### 3.3 The social system

#### 3.3.1 Management

##### Human management

Anthropogenic factors can be grouped into negative drivers such as draining, agricultural intensification, and fertilizing, and positive drivers like grazing and mowing (Table 1). The negative factors have for centuries changed the dynamic wetlands into areas suitable for modern agriculture. This has affected the key ecological patterns and processes of importance for wader population sustainability. Many earlier studies have identified these changes over Europe (Cronert, 2010; Emanuelsson, 2009; Manton & Angelstam, 2019). The most important negative driver is draining. The need for more effectively used arable and forestry land and the change of agricultural methods from the end of the 18th century increased draining (Gadd, 2000; Manton & Angelstam, 2018). Overall, this implies a transition from the creation of wader habitat as a side effect of sustaining local livelihoods based on animal husbandry, to active top‐down public sector biodiversity conservation which is often disconnected from farmers on the ground. For some farmers, however, experts reported that habitat management has become a main income through subsidies and tourism activities. Another major influence on breeding waders is the timing of mowing grasslands (Schroeder et al., 2012).

##### Wetland restoration

Restoration of wetlands for wader population sustainability requires a system perspective that encompasses entire landscapes and also includes the social‐ecological processes. Restoration of wet grassland therefore includes cutting shrubs and trees, introducing grazing and browsing herbivores, and mowing and re‐wetting drained areas to secure the water fluctuation capacity. Restoration methods based on historical knowledge have been tried and analyzed for the Swedish case study landscapes Östergötland and Öland. Methods like tree cutting, mechanic mowing and rotary cultivation to control tall grass vegetation outside the breeding season have been successful to restore the open wader breeding areas and grazing with different kinds of cattle upholding the long‐term sustainable wet grassland ecosystem. In the Örebro case study, landscape grazing today is dependent on agro‐environmental subsidies to the farmers.

##### Predator control

Methods for predator control can either decrease the number of predators or eliminate all kinds of predator food resources. In the Öland case study wader protection by shooting predators all year round since 2006 has been used to effectively decrease predator numbers with a positive effect on the wader population (Ottvall, 2015). The predator species included in the protection shooting are corvids, and badger (*Meles meles*), fox, marten (*Martes martes*), and mink (*Neovison vison*). The conservation focus on single threatened predator species without a systems perspective increases the intensity of predation on adult waders. One example is the introduction of artificial nest boxes for peregrine falcons (*Falco peregrinus*) and kestrel (*Falco tinnunculus*) (Svahn, 2016). Successful conservation of red kite (*Milvus milvus*) is another example (Manton & Angelstam, 2021; Manton et al., 2016). According to one of the experts, this insight led to canceling the introduction of peregrine falcon nest boxes in one case study.

##### Recreation

In all three central Swedish case study landscapes, the main recreation activities in the wetlands are birdwatching and fishing. The fishing associations support wetland restoration due to the open water for fish, especially pike (*Esox lucius*). Fishing possibilities also increase the landowners’ interest in wetlands. Wetlands as a green infrastructure close to urban areas are also identified to create social benefits through outdoor activity. For instance, Beery and Jönsson (2015) showed that the Kristianstad Nature Centre plays an important role by providing nature inspiration to its visitors to use and experience the wet grasslands of the Kristianstad Vattenrike Biosphere Reserve.

#### 3.3.2 Governance

##### Wetland restoration

To maintain or restore wader populations, wetland restoration has been attempted in several of the case study *landscapes*. In Sweden evaluations of restoration projects have found that, from a governance point of view, successful wetland restoration requires knowledge about wetland grassland history (Manton & Angelstam, 2018), and sustaining multiple synergies (Manton et al., 2016). This includes not only grazing and mowing but also a functional landscape‐level habitat network with meadows and open water (Jönsson, 2015). Pumping of water to modify the water level, which also benefits pike reproduction, and predator control are other good examples (Karlsson, 2017; Ottvall et al., 2008; Wallin et al., 2009). From a systems point of view, the single focus on wetland management may thus be detrimental as the management of the surrounding agricultural landscape is of crucial importance for successful wader conservation. The current homogenisation and afforestation of the wider landscape promoting predator populations is in this respect a challenge (Johannesdottir et al., 2019; Ottvall et al., 2008). Therefore, the tendency to prioritize management efforts only to areas with formal protection is problematic (Bergner, 2013).

##### Subsidies and landowner interest

Hansson et al. (2012) identify subsidies to farmers and local environmental benefits as drivers for a stakeholder interest in wetlands. Indeed, environmental policy subsidies have become a new source of major income to farmers for restoration and maintenance of wetlands through re‐wetting and grazing, which is a key factor for creating positive conditions for sustainable wader populations. Individual farmers have thus transitioned from providers of food to keepers of grazers that maintain wader habitat. However, the EU Common Agricultural Policy's focus on increasing agricultural production can be counterproductive, such as extensive drainage activities of drained organic soils (Manton et al., 2021).

### 3.4 Systems analysis

#### 3.4.1 Theme 1: Landscape ecology, wader bird breeding success

**FIGURE 4:**
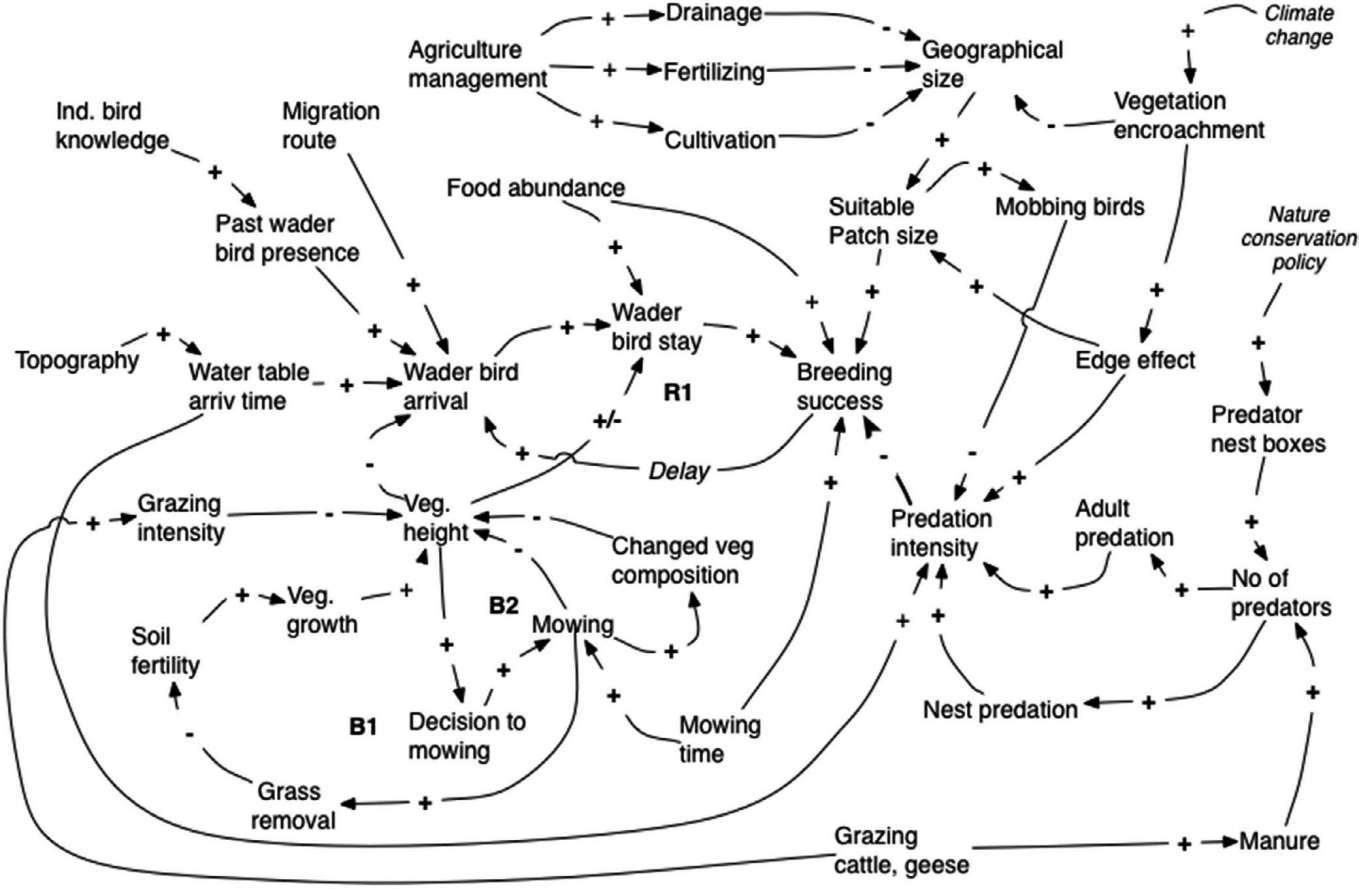
The local ecological system of wader breeding success and population development. Global/national outside drivers are given in italic

The first CLD model focused on the breeding success of waders (Figure 4). The predator factors have been combined in one group called “predator intensity” in order to allow the inclusion of both predation of nests, chicks and adults (Manton & Angelstam, 2021). A higher number of predators increase both adult predation and nest predation, which impact breeding success. The decision to introduce protective culling, i.e., shooting and trapping predators, is a regional outside management driver (Fletcher et al., 2010). Relatively few loops are identified in the ecological system while many drivers from outside the wet grassland system seem to be important. The main outside drivers on global and national levels were “nature conservation policy” and “climate change,” which according to experts increases vegetation encroachment and thus predation due to increased availability of perches for predators.

**FIGURE 5:**
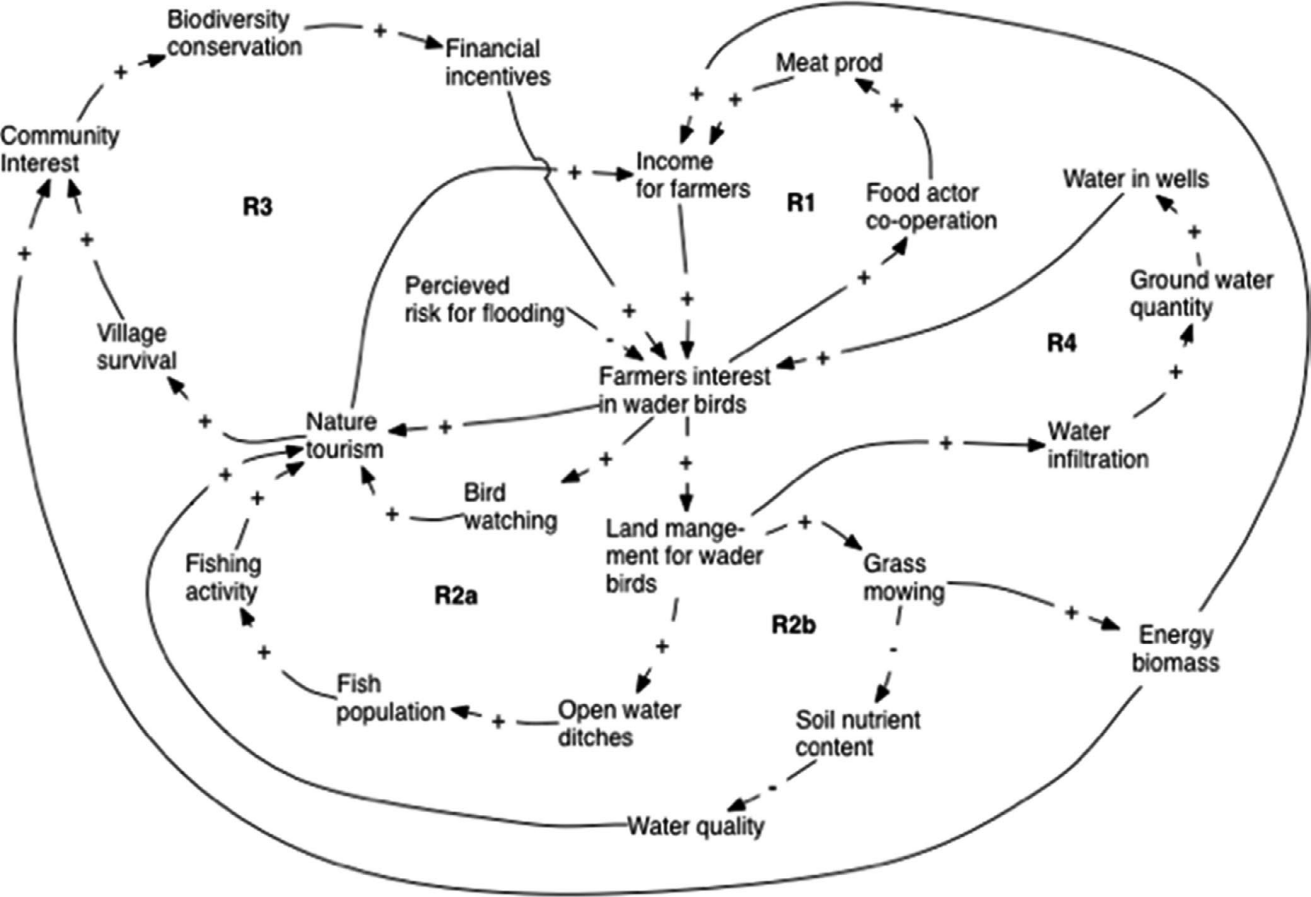
The management system of wet grasslands for wader birds. How to increase the farmer's interest

On the local scale the drivers “Land management” and “Vegetation encroachment” affect breeding success through “suitable patch size” and “predator intensity.” Predation can reduce the effective patch area. For example, within grassland patches, perches on wet grasslands increase predation risk. Conversely, a large patch size with more mobbing birds will reduce predation risk. For example, lapwings actively defend themselves against predators, and therefore other waders tend to breed close to lapwings and have somewhat higher breeding success. The “Land management” driver is further analyzed in the model for farmers’ interest in waders, see Figure 5. The driver “grazing” by cattle and population growth of geese (Tuvendal & Elmberg, 2015),.which regulates the grazing intensity is also linked to local land management (Sabatier et al., 2010).

One reinforcing and two balancing loops are found in the ecological wader system. The term “wader bird arrival” explains the number of waders deciding to the prospect on a certain geographic area. The population loop, R1, indicates that more arriving waders lead to a higher wader density (settlement after initial spring prospecting) and higher breeding success, which positively influence a higher wader bird arrival in subsequent years (increased recruitment). Another internal driver is the water table at the time of arrival. The two balancing loops, B1 and B2, depend on the farmers’ decision to mow. Mowing causes a change in vegetation composition and less vegetation growth due to nutrient output. Both loops result in low vegetation that increases the wader bird arrival.

#### 3.4.2 Theme 2: Optimal land management for wader birds

The second CLD modeling theme was the dynamics behind farmer's interest in wet grassland management for wader birds (Figure 5). Two reinforcing loops, R1, R2, increase the income of the local farmers and their interest in land management positive for wader birds. The third loop, R3, is the financial incentive loop triggered by an increased interest of municipality decision‐makers in biodiversity conservation and therefore a leverage point to wetland management on a landscape scale. The fourth loop affects the amount of water in the farmers’ wells.

The three income loops work together but the time when they become effective can vary. The leverage point in R3 starts the process on a landscape scale. As many of the wet grasslands are already grazed today, the prerequisite for R1, the food loop, exists. Meat production from natural grazed wetland, increases the “Farmer's income*”* and therefore the “farmer's interest in waders*”* and a higher local “Food actor co‐operation.” A higher co‐operation not only between farmers, but also with actors in the tourism sector, results in higher meat production through the number of animals grazing the land. More intensive grazing is a factor in the ecological system, decreasing the vegetation height and increasing wader bird arrival, i.e., the willingness of waders to choose a specific wetland. The nature tourism reinforcing loop, R2, says that a higher amount of “Nature tourism” increases the “Farmer's income,” the “Farmer's interest in waders,” and the *“*Land management for wader birds.” Increased “Land management” can increase “Nature tourism” either through wetland management for fishing tourism, R2a, or higher water quality through increased grass mowing and less nutrient in the soil, R2b. Land management suitable for waders also increases the number of bird watchers. A potential grazed land with high biodiversity starts the financial incentive loop, R3, which includes the following relationships: more “Income incentives” increases the “Farmer's interest in waders*”* and gives a higher amount of “Nature tourism” leading to a higher *“*Village survival,” which increases the “Community interest” for “Biological conservation” (for wetlands) resulting in more “Financial incentives” from the municipality.

#### 3.4.3 Theme 3: Society's interest in wetlands

**FIGURE 6:**
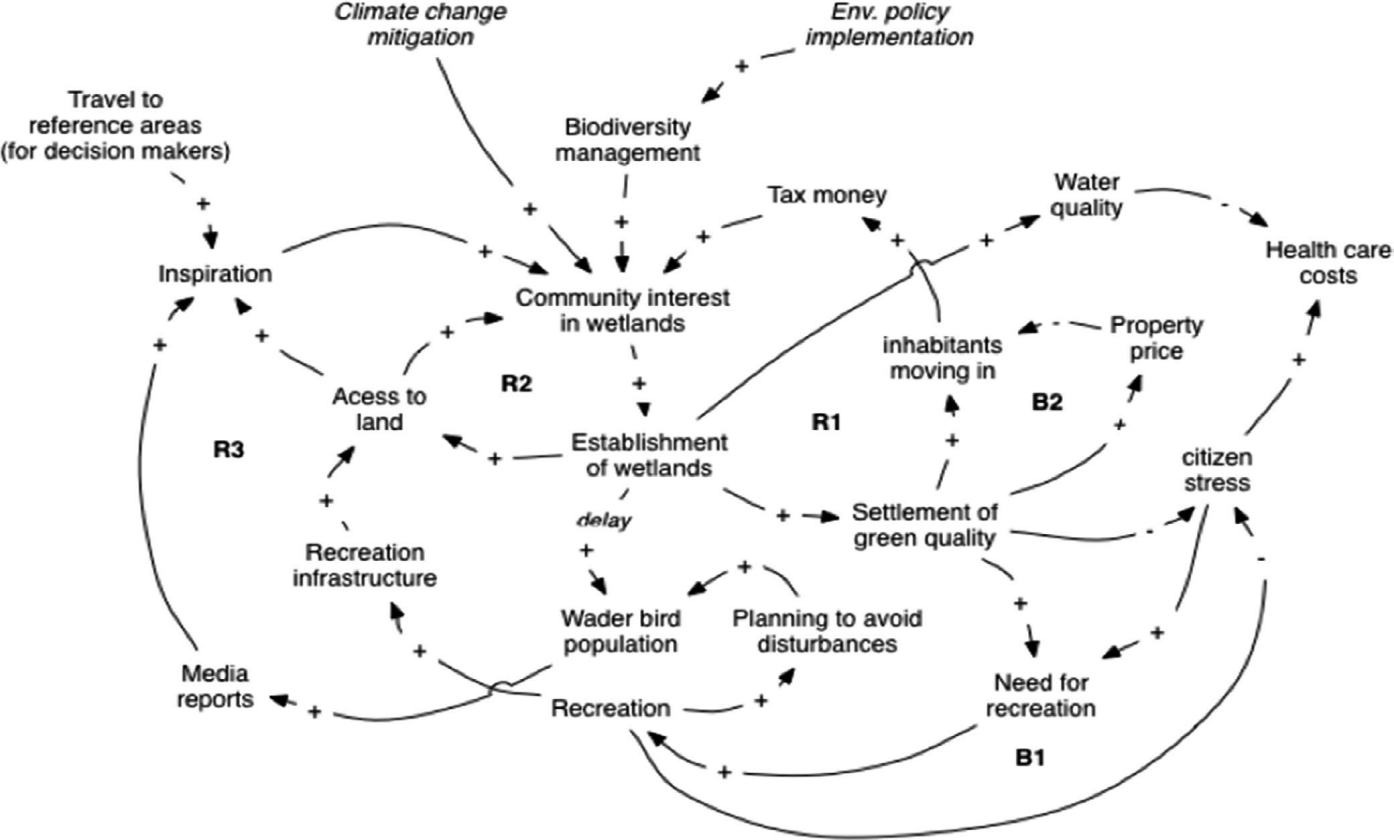
How to increase the community's interest in wader birds? Global/national outside drivers are given in italic

Two external drivers on the national level start the running of the loops through “Community interest in wetlands.” Community includes public sector decision‐makers in society. Additional objectives include the need for climate change mitigation using the wetland as a CO_2_ sink and the environmental policy to keep and increase biodiversity. The variable “Community interest wetlands” is also included in the R3 loop in Figure 6. In the community interest system, there are three reinforcing loops and two balancing loops. All the reinforcing loops go through the “Community interest in wetlands.” The income loop, R1, starts when the community develops a higher interest in establishing wet grasslands forced by the external drivers. The greening of the area results in more *“*Settlement of green quality*”* and a higher *“*Tax income*”* increasing the “Community interest in wetlands.” There may be a multi‐year lag between establishment of wetlands and the settlement of green quality.

The loop access to land for recreation, R2, starts as soon as a higher “Community interest in wetlands” gives more “Establishment of wetlands,” which gives a higher “Access to land” for tourists and citizens which results in a higher “Community interest in wetlands.” The Inspiration loop, R3, focus on that a higher “Establishment of wetlands” – gives a higher “wader bird population” through land management and therefore a higher amount of “Media reporting,” which gives more “Inspiration” to the community politicians and a higher “Community interest” in wetlands. This loop has a delay as it takes time to increase the wader population after the establishment of suitable wetlands.

This system also has two balancing loops. The first, B1, tells us that “More green settlements” gives a higher “Need for recreation” and a higher amount of “Recreation activity.” More recreation activity results in less “Citizen stress” and less “Need for recreation.” The other balancing loop, B2, the property price loop, hampers the amounts of citizen moving into the area due to the increasing cost of housing.

The ecological system in Figure 4 is linked together with the social system in Figures 5 and 6 through the wetland management and community interest concepts. Figures 4 and 5 are linked through “Grass mowing” and “Soil nutrient content” and Figures 4 and 5 through community interest in wetlands. Higher breeding success of wader birds in the ecological system, Figure 4, increases wader populations in the community interest system. The global external driver, climate change, will affect both the ecological system, as described in Figure 4, and the community interest system (Figure 6).

### 3.5 Validation in space and time

For validation of the systems analysis in the current study, we chose to discuss the results in the contexts of two landscapes characterized by a historical focus on animal husbandry and the establishment and maintenance of grasslands with different levels of wet grassland conservation success. These were Kristianstad Vattenrike in southern Sweden, which has been unsuccessful in terms of conserving wader populations (see Manton et al., 2016), and Friesland in the Netherlands, which has been effective at the local level (Kentie, 2015). For details, see Appendix S2.

Using historical maps and agricultural statistics of Kristianstad Vattenrike, Manton and Angelstam (2018) analyzed the process of grassland deterioration over 200 years owing to changes in agriculture management, land consolidation leading to intensified crop production and changed economical demands. Besides the accumulated loss of grassland area, fragmentation, changes in patch size and hydrology, predation, and edge effects were identified as factors impacting negatively the functionality grasslands as a green or ecological infrastructure. The social driver was changes in animal husbandry from low intensive farming using extensive pastures to intensive farming. These factors and their cause‐relationship may similarly be identified in the CLDs in Figures 4 and 5, thus supporting the models. To increase the farmer's interest in waders and wetlands new long‐term income sources ought to be developed. Figures 4 and 5, the farmers’ and communities’ interest in wetlands show the economical possibilities of how to return to extensive grazing and mowing through tourism, recreation, and high‐quality meat production.

Dominated by agricultural areas with high wader bird conservation value Friesland in the Netherlands was epitomized by the term “'meadow birds.” Supporting the notion of wet grassland development stages (Figure 3), already several decades ago swamps and bogs were transformed into meadows and pastures (Van Gijn & Waterbolk, 1984). The ca. 75% decline of godwits since the 1960s coincides with agricultural intensification based on artificial fertilizers, land consolidation creating larger grassland patches, lowered ground water, drainage pipes below ground instead of open ditches, and more productive grass species (Harms et al., 1987). To cope with this, conservation of meadow birds is attempted by avoiding disturbance, not using fast‐growing grass species, increasing water table, leaving grass uncut around nests, and putting a metal frame over it. However, according to Montfoort (2020) targets for Friesland are unlikely to be achieved without substantial changes in supportive systems for farmers to participate in nature conservation and restoration measures. To conclude, as in the case study wetlands used for systems analysis (Figure 6), the social system dimensions are neglected in Friesland.

## 4 DISCUSSION

### 4.1 Systems analysis as tool for knowledge production and learning

As there are numerous factors that, simultaneously but with varying time lags, may affect the functionality of green infrastructures the frequent lack of systems perspective in biodiversity conservation policy is problematic (Harvey et al., 2017). Viewing a wet grassland landscape as a social‐ecological system highlights the complex interactions among multiple factors and the dynamic and nonlinear characteristics of processes involved. Systems analyses of landscapes as social‐ecological systems can support collaborative learning in individual wet grassland landscape by diagnosing the state and trends of land cover types as green infrastructures, and setting priorities for conservation, management, and restoration.

Successful management of ecological systems includes re‐wetting, mowing, and grazing by cattle and horses in sufficiently large patches of wet grasslands, and predator control (McMahon et al., 2020). This is confirmed in several wetland landscapes other than the ones we analyzed (Kuresso & Mägi, 2004). In Estonia, Rannap et al. (2017) found that extensive grazing and no woody vegetation were positive for wader breeding conditions. Located in the East‐Atlantic flyway for migrating birds the Matsalu National Park is situated around a river delta and the coastal grasslands are maintained using a combination of low‐intensity grazing and mowing regimes to create an open sward. The Dviete floodplain in south‐eastern Latvia on the left bank of the Daugava River was managed by mowing and grazing until the mid‐20th century (Gruberts & Štrausa, 2011). During the Soviet period, collective farming was introduced with mechanized cultivation of grasslands on drier soil, and wet meadows were abandoned. Conservation management projects have restored open grassland by clearing vegetation and introducing cattle and Konik horses, and restoring 2 km of natural river meanders (Ķerus et al., 2015).

Predation on nests and chicks is an example of links between the dynamics of ecological and social systems (Laidlaw et al., 2021; Manton & Angelstam, 2021). Comparing five of the wet grassland landscapes in this study Manton et al. (2019) found that corvid bird abundance, and availability of their resources, increased with increasing agricultural land‐use intensity. This is consistent with the increase in abundance of the corvid species in southern Sweden over the past few decades (Ottvall et al., 2009), especially in a mixed mosaic landscape of agriculture and forest (Andrén, 1992). Also, the abundance of raptors has changed. In their analysis of raptor observations at the Danish Tipperne wet grassland case study 1930–2011, Meltofte and Amstrup (2013) conclude that almost all raptor species have increased due to multiple drivers. First, persecution of raptors decreased considerably until they gained full protection in 1967. Second, environmental pollutants peaked in the 1960s and 1970s, but later, raptor populations gradually recovered. For example, Thorup and Bregnballe (2015) showed that the presence of peregrine falcons (*Falco peregrinus*) at Tipperne in the wader breeding season is very much more frequent in the past 15–20 years than observed in any previous periods. Third, emergence of conifer plantations during the 20th century likely improved the conditions for sparrowhawk (*Accipiter nisus*), goshawk (*Accipiter gentilis*), common buzzard (*Buteo buteo*), and kestrel (*Falco tinnunculus*). Fourth, meadow management has changed. Initially, meadows were intensively used for mowing and grazing, which are negative factors for important predator‐prey species such as water vole (*Arvicola terrestris*) and field vole (*Microtus agrestis*). Controlling predators can thus be effective. Beginning in 2008 culling and trapping of mammalian (fox, badger) and avian (corvids and gulls) predators was carried out in the Swedish Öland wet meadow landscape (Karlsson, 2017; Ottvall, 2015). Early spring culling was most effective to increase chick survival of black godwits. Corvids had only a minor impact on nest predation (Ottvall et al., 2009). The role of predators has been highlighted also in other systems. Regarding recent declines in Norwegian seabird populations, Fauchald et al. (2015) concluded that apart from ecosystem changes affecting the availability of prey, increased predation is from avian and mammalian predators is a key factor. Especially for declining and threatened populations, this stressor is particularly important to control. Thus, increased predation on seabirds is an unintended consequence of the recovery of sea eagle *Haliaeetus spp*. populations (Hipfner et al., 2012). In a natural experiment, Hentati‐Sundberg et al. (2021) confirmed this and also reported a previously concealed guarding effect by tourist groups on an iconic seabird colony in the Baltic Sea. Triggered by the COVID pandemic, a halt of visiting tourists in 2020 led to a strong increase in presence of white‐tailed eagles but facilitated egg predation from herring gulls (*Larus argentatus*) and hooded crows (*Corvus cornix*). Thus, a social‐ecological systems perspective is crucial for successful seabird management.

The main leverage point in the social systems is the farmer's interest in wetland management which is triggered by financial income loops. In the absence of livelihoods based on grasslands as a driver to maintain suitable land covers for waders, adequate, long‐term economical subsidies form the basis for wetland establishment and grassland maintenance (Gren et al., 2021; Hansson et al., 2012). However, an unfavorable social‐ecological driver that these areas do not provide is a long‐term steady income based on production, but income is dependent on short‐term subsidy programs for restoration (Borgström et al., 2016). As the governmental subsidy system is complex, not transparent, and short‐term, the identified local income reinforcing loops are fundamental for grassland management. The loop including meat production (R1, Figure 5) is in line with the growing interest for high‐quality meat from grazed semi‐natural grasslands (Emanuelsson, 2009). To secure a long‐term economic stability for maintenance, Gren et al. (2021) even suggested a climate tax on food consumption with refunding to farmers for ecosystem services. Outdoor recreation and nature‐based tourism are other sources of income (Margaryan & Fredman, 2017). Wet grassland restoration in urban settings has led to increasing housing prices and the increased green infrastructure to a perception of better human health and well‐being (Stoltz, 2020).

Complex social‐ecological problems such as maintenance of green infrastructure are frequently handled from a top‐down silo perspective with attendant poor coordination between different government agencies and often conflicting advice or decisions further reduce legitimacy at the local society level (Schlyter et al., 2013). National nature and landscape conservation policy tend to lack a systems perspective (Borgström et al., 2016). This applies not only to the ecological landscape dynamic, which is demonstrated by the initiative to put up predator nest boxes close to wader wetlands (Thorup & Bregnballe, 2015) but mainly by not recognizing the social and livelihood aspects of farming and grassland management (Raatikainen & Barron, 2017). This is especially evident with regard to subsidies for wetland establishment and management in Sweden.

New modes of governance to satisfy complex environmental objectives on EU, national and local levels identified deliberative methods and dialog as important for a positive outcome (Bäckstrand et al., 2010). Wetland projects with a positive result for nutrient retention and biodiversity development are often landowner driven, where the transformation of practical knowledge and adaptive learning is a prerequisite for effective measures (Hansson et al., 2012). However, in contrast, authorities often devise top‐down administrative processes, seen as safer for reaching environmental objectives as opposed to bottom‐up stakeholder‐based approaches. For instance, Birge et al. (2017) found that in Finland, administrative officials were opposed to a suggested result‐oriented payment scheme for grasslands, owing to their perceptions that this approach does not fit into the current institutionalized program.

In the future, global warming will increase temperature and change the precipitation pattern, thus affecting the local ecosystems’ water and vegetation drivers but also social drivers as the local community's awareness of changes in the landscape and the need for mitigation. The identification of wetlands as possible CO_2_ sinks (Manton et al., 2021), and flood mitigation (Barbedo et al., 2014) call for long‐term management through extensive low intensive grazing (Benstead et al., 1997), which is only possible with a continuous income from farming and tourism or environmental subsidies. The driver to establish more wetlands for waders at the community level is connected to biodiversity conservation, a growing social interest in settlements with “green” qualities and landscape recreation, and climate change.

### 4.2 Placing landscape case studies in the systems analyses

The eight different case study landscapes which formed the empirical base for the systems analysis face a wide range of challenges and opportunities for sustaining wet grasslands as a green infrastructure for biodiversity conservation and human well‐being. Additionally, the histories of two wet grassland landscapes were used for validation. Representing a long gradient from favorable to unfavorable social‐ecological conditions for wader bird populations (Figure 3), below we link the systems analysis to these 8 wet grassland landscapes (Table 2).

**TABLE 2:** Overview of barriers (−) and bridges (+) for wader bird conservation in eight wet grassland landscape case studies

	Theme 1: Landscape ecology and breeding bird success	Theme 2: Optimal land management for wader birds	Theme 3: Society's interest in wetlands
Floi/Eyrarbacki	+ Grasslands, along with sedge meadows, are dominant land covers	+ Management for animal husbandry deliver habitat	+ Vital economic resource + Agricultural subsidies
Turov	+ Grasslands are a dominant land cover	+ Traditional management for animal husbandry delivers habitat	+ Vital economic resource + Conservation
Tipperne	+ Grassland patches are large	+ Predator control − Organic/animal welfare grazing procedures force too early grazing	+ Conservation, recreation
Öland	+ grassland patches are large	+ predator control + continuous grazing and mowing + re‐wetting	+ conservation, recreation
Nemunas delta	+ grassland patches are large	− Grazing and mowing are declining	+ Conservation + Economic resource
Mälardalen	− Small patches	+ Predator control + Continuous grazing and mowing − Intensive use of the surrounding landscape	+ Conservation, recreation
Östergötland	− Small patches	+ Agricultural subsidy + Predator control + Continuous grazing and mowing. − Intensive use of the surrounding landscape	+ Conservation, recreation
Kristianstad	− Small patches − Generalist predators	− Intensive use of the surrounding landscape	+ Conservation, recreation

The initial ranking placed the Kristianstad Vattenrike Biosphere Reserve in southernmost Sweden as an extreme case among the wet grassland case study landscapes. Comparative studies of wet grassland landscapes show that multiple unfavorable factors for biodiversity conservation are concentrated in the Kristianstad Vattenrike grasslands. Here the land covers contributing to the wet grassland infrastructure have declined from the past by two orders of magnitude (Manton & Angelstam, 2018) during the past two centuries. Bird surveys made in this part of Sweden in the 1930s show that currently extirpated wader bird species like ruff and dunlin were previous regular breeders (Jönsson et al., 2021). Today, the remaining grassland patches are small, the quality is declining, and generalist predators are abundant. Nevertheless, this area has repeatedly been presented as a success story of environmental governance and adaptive co‐management of green infrastructure (Millennium Ecosystem Assessment, 2005; Olsson, Folke, et al., 2007; Olsson, Schultz, et al., 2007; Schultz et al., 2015). In spite of this, restoration efforts have been short‐lived and the conservation status of the priority green infrastructure being semi‐natural grassland ecosystems with an unfavorable situation for wader birds has remained (Cronert, 2010, 2014; Manton & Angelstam, 2021). This places Kristianstad in the past and at present at two extremes and illustrates the role of agricultural intensification (Johannesdottir et al., 2019). Two other case study landscapes in Sweden (Östergötland and the Mälaren area) have similar problems (Table 2; Manton & Angelstam, 2021; Manton et al., 2016, 2019). On the other hand, today, the role of these wet grassland areas for recreation and nature‐based tourism has become increasingly important (Beery & Jönsson, 2017; Beery et al., 2017). This is in clear contrast with the past when wet grasslands were an important resource for local and regional livelihoods linked to animal husbandry.

The Lithuanian Nemunas Delta has transitioned between different land‐use systems. Initially, traditional small‐scale farming based on animal husbandry prevailed until World War 2. Subsequently, Soviet occupation ensued, and land use changed land covers, which transformed to industrial farming. After Lithuania regained independence, this was followed by complex patterns of land abandonment, animal husbandry, and intensified agriculture. Rural exodus is currently leading to reduced grassland management. The Danish Tipperne is one of the oldest nature reserves in Denmark, and land use has been conservation orientated for almost 100 years. Meadows are maintained by a combination of organic cattle grazing procedures and mowing, just like the Öland case study.

At the other end of the gradient of contemporary green infrastructure functionality, the case study landscape in Belarus has by and large maintained traditional and semi‐natural grassland systems, once widespread in all case study landscapes. Being large and stable the Turov wet grassland landscape has viable populations of waders and is used as a benchmark to produce knowledge for wet grassland areas in other countries (Benstead et al., 1999).

Finally, in Iceland positive factors dominate. Agricultural subsidies are a key factor that supports the maintenance of grasslands for cattle breeding and dairy production (Johannesdottir et al., 2019). This results in the maintenance of a cultural landscape with grazed wetlands (where sedges dominate over grasses), which are used by the same species as what is termed wet grasslands elsewhere. Combined with large heathland and grassland areas for nesting this provides important habitats for feeding (Johannesdottir et al., 2019).

Our attempt to validate using the histories of wet grassland landscapes in Kristianstad and Friesland confirms the comparative approach. Long‐term changes in land use intensification and trophic interactions have caused declines of species dependent on wet grassland landscapes in both areas and have triggered considerable research efforts. However, researchers’ focus on disciplinary versus integrative research affects the conclusions, thus, although Kristianstad conservation of wet grassland has made humans benefit through outdoor recreation (Beery & Jönsson, 2015, 2017), the wader birds have not (Manton et al., 2016). Coordination and integration of social system actors is often a limiting factor for successful wader bird conservation (Montfoort, 2020), and not lack of ecological knowledge.

### 4.3 Multiple landscape case studies as a tool

Implementing policy on green infrastructure requires evidence‐based knowledge about the states and trends in terms of biodiversity conservation and provisioning of ecosystem services, which is combined with cross‐sectoral multi‐level environmental governance. This implies a social‐ecological systems approach (Herzon et al., 2021). Maintaining wet grasslands as a green infrastructure is more than just about managing land covers and needs to involve many factors at multiple spatial scales. A functional green infrastructure is not only about patch quality linked to hydrology, grazing, and mowing; patch size; and functional connectivity of acceptable patches (Manton & Angelstam, 2018). It is also trophic interactions such as predation (McMahon et al., 2020), for example on nests and chicks by corvid birds (Manton & Angelstam, 2021). The values of wet grasslands are co‐generated by interacting social and ecological systems and linked to traditional land‐use practices aimed at producing food and feed. To replace these disappearing practices agro‐environmental schemes have been developed with the aim to maintain biocultural values as the “key products.” This has indeed provided support for grassland landscapes as sites for outdoor recreation (Beery & Jönsson, 2015), which is much less complicated than to secure functional green infrastructure for biodiversity conservation. Thus, land management and governance should be developed with a better awareness of the challenges linked to different benefits provided by wet grassland landscapes as social‐ecological systems. The implementation of specific policy instruments to financially support land managers to supply values to society represents a future challenge that both research and policy makers should focus upon (de Groot et al., 2010). Second, both anthropogenic and natural processes from individual land cover patches through to landscape and regions need to be understood. Hence, the conservation of semi‐natural grasslands as functional green infrastructure is complex (Benstead et al., 1997) and requires continuous knowledge production and learning, and ongoing maintenance and monitoring programs to assess consequences on the ground (Rauschmayer et al., 2009). The systems analysis approach to enhance collaborative learning among researchers and stakeholders through analyses of multiple landscape case studies is an appropriate tool for practicing transdisciplinary research through collaboration among natural and social scientists and practitioners in different contexts.

Increasing demands for natural resources, an exodus from rural regions, biodiversity conservation, and climate change require environmental governance systems that can exercise transformative change towards sustainable landscapes. This requires evaluation of policy implementation in terms of what develops between the establishment of an agreed policy and the ultimate impact of subsequent actions in the real world (Rauschmayer et al., 2009). Three key evaluation steps are (1) the policy process (e.g., who takes part?), (2) outputs (e.g., policy instruments, planning processes?), and (3) the consequences in terms of outcomes on the ground (e.g., the functionality of ecological networks, or green infrastructures, forming trust, livelihood for landowners, supporting human well‐being and biodiversity conservation). Given that evaluation methods need to recognize that restoration is driven by multiple rationales (Baker & Eckerberg, 2016) the outcomes on the ground take a long time to develop, an alternative is comparative macroecological comparative studies based on multiple place‐ and area‐based case studies representing different trajectories of land use and land cover change and social‐ecological systems can be seen as “landscape experiments” is another alternative.

## CONFLICT OF INTEREST

None of the co‐authors have any conflict of interest.

## AUTHOR CONTRIBUTIONS

**Per Angelstam:** Conceptualization (lead); Data curation (equal); Formal analysis (equal); Funding acquisition (lead); Investigation (lead); Methodology (lead); Project administration (lead); Resources (lead); Validation (lead); Visualization (equal); Writing – original draft (lead); Writing – review & editing (lead). **Michael Manton:** Conceptualization (equal); Data curation (equal); Formal analysis (equal); Funding acquisition (supporting); Methodology (equal); Validation (equal); Writing – original draft (equal); Writing – review & editing (equal). **Ingrid Stjernquist:** Conceptualization (equal); Formal analysis (equal); Methodology (equal); Writing – original draft (equal); Writing – review & editing (equal). **Tómas Grétar Gunnarsson:** Validation (equal); Writing – review & editing (equal). **Richard Ottvall:** Data curation (equal); Writing – review & editing (equal). **Mats Rosenberg:** Writing – review & editing (equal). **Ole Thorup:** Data curation (equal); Validation (equal); Writing – review & editing (equal). **Per Wedholm:** Writing – review & editing (equal). **Jaanus Elts:** Writing – review & editing (equal). **Davis Gruberts:** Data curation (equal); Writing – review & editing (equal).

## Supporting information

Appendix S1‐S2

## Data Availability

Data are available in Table 1, in the two Appendices; verbal data for causal loop modeling were not recorded as part of the analytic process.
