# Supplementary material for: Barriers and bridges for sustaining functional habitat networks: A macroecological system analysis of wet grassland landscapes
Source: Ecol Evol. 2022 Apr 6;12(4):e8801. doi: 10.1002/ece3.8801 (PMC8986984; doi:10.1002/ece3.8801)
Supplement: Supplementary file 1 — Appendix S1‐S2 [file ECE3-12-e8801-s001.docx]

# Appendix 1. Case study landscapes used for CLD modelling

## Floi reserve (Iceland)

The Floi nature reserve (ca. 63°N, 21°W) is situated on the eastern bank of the estuary of the River Olfusa in southern Iceland (Figure 1). The ca. 500 ha area is a wet sedge meadow at 2 m a.s.l with numerous small ponds. The Floi reserve is only a small a part of extensive flooded wetlands, which used to cover the coastal plains of south Iceland. Beginning in the 1940’s resulted in over 90% of wetlands affected by draining. The Icelandic Birdlife partner Fuglavernd joined forces with the local Eyrarbakki (now Árborg) municipality council in 1997 to start wetland restoration in the Floi reserve. Most of the drainage ditches in the reserve have now been filled in. In recent years, infrastructure for visitors has been built, including a birdwatching hide and paths through the reserve. Several wader species breed in the area in high density along with various species of water birds (https://fuglavernd.is/habitats/floi-bird-reserve/). Habitat management in the reserve is negligible. Some uncontrolled grazing occurs in the reserve, but local farmers release a small number of sheep and horses there every summer which help maintain suitable sward heights for breeding waders. Some trapping of mink (*Neovison vison*) occurs in and close to the reserve, but otherwise there is no management of predation.

## Tipperne (Denmark)

Established as a nature reserve in 1928, Tipperne (ca. 55°N, 08°E) comprises part of a peninsula in westernmost Denmark (Figure 1) with brackish wet meadows, reeds and sand dunes surrounding the shallow water of Ringkøbing Fjord (Gravesen 1972, Jacobsen 1975). Tipperne is a unique due to its long history of bird and habitat monitoring (e.g., Laursen et al. 2008, Meltofte 1987, Olsen and Schmidt 2004, Thorup 1997) Hydrology practises have changed with shifting aims. For decades, salinity was kept low year-round, but since 1995 the summer salinity was increased substantially. Sedimentation gradually increased the land area from approximately 485 ha in 1928 to 665 ha in 2015 (Thorup 1998, 2003, 2016). Hay harvest and cattle grazing on the grasslands is widespread, only interrupted from the late 1950s till the early 1970s, which led to spreading of reed (*Phragmites communis)* (Møller 1975, Thorup 1998). In the mid-1970s hay making/mowing and cattle grazing were reintroduced to re-establish short-grass wet grasslands without reed fringes with adjacent mudflats (Pakanen and Thorup 2016). Breeding conditions of waders are much more favourable in wet than in dry springs (Thorup 2016). Since 1928, observers have aimed at annually recording the breeding birds present in the Tipperne scientific reserve that today covers 511 ha of grazed and mown grassland and 90 ha of grazed grass wetland (Thorup 2016). The Tipperne grassland unit also includes the adjacent Værnengene grassland (1150 ha) (Thorup 2003).

## Öland (Sweden)

With its shallow soils on calcareous bedrock, the Swedish island of Öland in the Baltic Sea (Figure 1) hosts barren alvar and coastal grasslands where the water supply, owing to the karst environment, fluctuates considerably. Because the island is slightly tilted eastwards, most coastal grasslands are found on the eastern shores. Of the seven protected grassland areas, the Sydöstra Ölands Sjömarker Wetlands is a 9000 ha, 28 km long, World Heritage area making it one of the larges coastal grasslands in Europe (ca. 56°N, 16°E). The area has a grazing tradition since the Iron Age and is an important migratory and nesting area for waders. Through a LIFE-BaltCoast project some of the existing ditches have been blocked, to recreate wet habitats, with a positive effect on waders. Since 2007 the area has also been part of a long-term predation control project (Ottvall 2015) with the objective to improve the breeding success of waders (e.g. Ottvall 2005). In the southernmost part of the island and next to Sydöstra Ölands Sjömarker Wetlands is the 1026 ha Ottenby Nature Reserve (https://www.lansstyrelsen.se/kalmar/natur-och-landsbygd/skyddad-natur.html). The eastern part is open grassland with an undulating topography generating a mix of dry meadows and wet grasslands. The area has historically been managed by continuously mowing, but today only 55 ha is mowed, and the other part is grazed by sheep and cattle. Since 1980 the ground water level has decreased but Ottenby still remains an important area for resting and feeding of migratory birds.

## Kristianstad Vattenrike (Sweden) –present

Degradation of the wet grasslands around Kristianstad city (ca. 56°N, 14°E) can be traced back >200 years with three factors leading to their degradation: (1) agricultural intensification linked to land consolidation, (2) the planting of trees and forest, and (3) the lowering of the surface water table of the Helge å River by changing its natural course to the Baltic Sea in 1774 (Sjöbeck 1973). Manton and Angelstam (2018) showed that the combined loss, alteration, and fragmentation of Kristianstad’s semi-natural grassland as functional GI has declined by more than 98% during the past 2 centuries. This has led to a decline in wader birds. This applies in particular to the Dunlin which is no longer breeding in the area, the Ruff which only breeds sporadically, and the Black-tailed Godwit may soon follow. In addition, also more common species such as Lapwing, Redshank and Curlew (*Numenius arquata*) are declining, albeit at a lower rate (Manton et al. 2016). The importance of the wet grassland triggered the designation as a RAMSAR wetland area and the Kristianstad Vattenrike Biosphere Reserve (Magnusson et al. 2004). The wet grasslands are surrounded by agricultural land, urban areas, and encroaching shrublands and forests.

## Östergötland (Sweden)

The Östergötland landscape (ca. 58° N, 15°E) consists of one of southern Sweden’s few inland wet grassland systems with relatively stable breeding wader populations (Manton et al. 2016, Bergner 2013). This case study contains a cluster of three Ramsar wetlands i) Lake Tåkern, ii) Lake Roxen and iii) the Bråviken Bay and a variety of other wet grasslands. The areas consist of freshwater lakes and wetlands with adjacent shore meadows and pastures that vary in conservation status from favourable to unfavourable (Bergner 2013). Indeed, large parts of these grasslands are managed and have undergone restoration activities for the conservation of wader bird, which have been successful. Ongoing management strategies for wader bird conservation include modified grazing and mowing programs. Unlike the other Swedish case studies the landscape matrix in Östergötland is coarse-grained agricultural land. The area is an important breeding and stopover site for many wetland birds. Lapwing and redshank are the most common wader species breeding on the wet grasslands.

## Mälardalen (Sweden)

The Mälardalen landscape (ca. 59°N, 15°E) in south-central Sweden (Figure 1) is characterised by a transition of open agricultural fields in the south to predominantly forest in the north. The landscape mosaic is fine-grained with many landowners and contains small remnant semi-natural grasslands of traditional land use, all with a history of hay-making and grazing in the past (Palmqvist 1980). For example, until the late 19th century the agricultural plains in the lower reaches of the catchments of the two rivers Täljeån and Svartån near the city Örebro had a large proportion of wet grasslands used as pastures and meadows (Lennquist 2007). However, large areas of wet grasslands were lost when the adjacent lake Hjälmaren was lowered 1882–1886 (Rydin and Borgegård 1991) to gain agricultural land. Efforts to restore wetlands and wet grassland began in the 1990s (Olsson 2009). Recently much restoration work has been undertaken to reclaim overgrown wet grasslands for wader birds (Berg et al. 2002). Restoration efforts to maintain species related to traditional agricultural management (Lindborg and Eriksson, 2004) are supported by short term environmental schemes to undertake mowing and grazing.

## Nemunas (Lithuania)

The Nemunas river delta in Lithuania (ca. 55°N, 21°E) (Figure 1) consists of a maze of river tributaries exiting into the Curonian Lagoon and the Baltic Sea. The landscape consists of approximately 30000 ha of grassland, which naturally floods in late winter and early spring with water exceeding sea level by 3 m (Stanevičius et al. 2008). Due to annual flooding the first pump station built in 1907 and now contains 21 polders and 39 pump-houses. The Nemunas delta has a long history of grassland cultivation. During Soviet occupation land covers transformed from traditional small scale farming based on animal husbandry to industrial Soviet farming. However, when Lithuania re-gained independence in 1990 the region experienced high levels of land abandonment. Currently land management for intensification for both the forest and agricultural sectors is taking place, including the restoration of the polder system. The delta is farmed predominantly for dairy produce and fodder for livestock. With the landscape retaining much of its natural dynamics and hosting large numbers of migrating birds the delta was declared Ramsar wetland in 1993. However, declines in wader birds have been recorded over the last decades (Thorup et al. 2011).

## Turov (Belarus)

The mid Pripyat River Valley in southern Belarus’ (ca. 52°N, 27°E) (Figure 1) is one of Europe’s largest (950 km^2^) authentic wet grassland landscapes. The Pripyat River landscape contains large tracts of land covers that still hosts patterns and processes characteristic of both natural and traditional cultural landscapes (Benstead et al. 1999). The meandering Pripyat River has an extensive floodplain with an average width of 8-9 km. The floodplain contains many oxbows, ponds and low depressions that recharge during the floods. Land use includes grasslands for hay-making, cattle grazing and encroaching shrublands. In the surrounding landscape, land use centres on industrial agriculture run by state cooperative farms. All land is owned by the state, thus agricultural activities are undertaken at large spatial scales. During the past 30-40 years approximately 25 % of the floodplain has been drained. Nevertheless, the Pripyat River valley remains one of the most authentic wet grasslands in Europe and as a result is a key breeding and stop over area for many migratory bird species and has been declared an area of international importance (Pinchuk et al. 2005).

# References

Benstead, P., Drake, M., Jose, P., Mountford, O., Newbold, C., Treweek, J. 1997. The Wet Grassland Guide. Sandy, UK: Royal Society for the Protection of Birds.

Berg, Å., M. Jonsson, T. Lindberg, K.-G. Källebrink. 2002. Population dynamics and reproduction of Northern Lapwings Vanellus vanellus in a meadow restoration area in central Sweden. Ibis 144:131-140.

Bergner, A. 2013. Fåglar på strandängar i Östergötland - inventeringar 1996-2013. Länsstyrelsen Östergötland.

Gravesen, P. 1972. Plant communities of salt-marsh origin at Tipperne, western Jutland. Botanisk Tidsskrift 67:1-32.

Jacobsen, N.K. 1975. Zoneringsmodel for Det danske Vadehav med henblik på etablering af en dansk naturpark Tipperne—Skallingen—Vadehavet. Geografisk Tidsskrift-Danish Journal of Geography 74(1):68-69.

Laursen, K., Elmeros, M., Holm, T.E., Asferg, T., Amstrup, O., Bak, M., Thorup, O., Madsen, A. B., Hansen, T.S. 2008. Dømt på mistanke? Et prædations-projekt på Tipperne Dansk Ornitologisk Forenings Tidsskrift 102(2):246-247.

Lindborg, R., O. Eriksson. 2004. Historical landscape connectivity affects present plant species diversity. Ecology 85:1840-1845.

Lennqvist, J. 2007. Våtmarkshistoria: Hjälmarens och Kvismarens stränder under 1800- och 1900-talen. Örebro universitetsbibliotek, Örebro.

Magnusson, S.-E., Magntorn, K., Wallsten, E., Cronert, H., Thelaus, M. 2004. Kristianstads Vattenrike Biosphere Reserve Nomination Form; Springer: Berlin, Germany.

Manton, M., Angelstam, P. 2018. Defining benchmarks for restoration of green infrastructure: A case study combining the historical range of variability of habitat and species’ requirements. Sustainability 10:326. doi:10.3390/su10020326. <http://www.mdpi.com/2071-1050/10/2>

Manton, M., Angelstam, P., Milberg, P., Elbakidze, M. 2016. Wet grasslands as a green infrastructure for ecological sustainability: Wader conservation in Southern Sweden as a case study. Sustainability 8(4):340; doi:10.3390/su8040340

Meltofte, H. 1987. Forekomsten af rastende vadefugle på reservatet Tipperne 1928-1982. Dansk Orn. Foren. Tidsskr. 81:1-108

Møller, H.S. 1975. Danish salt-marsh communities of breeding birds in relation to different types of management. Ornis Scandinavica 6:125-134.

Olsen, H., Schmidt, N.M., 2004. Impacts of wet grassland management and winter severity on wader breeding numbers in eastern Denmark. Basic and Applied Ecology 5(2):203-210.

Olsson, J. 2009. The power of the inside activist: Understanding policy change by empowering the Advocacy Coalition Framework (ACF). Planning Theory & Practice 10(2):67-187.

Ottvall, R. 2005. Boöverlevnad hos strandängshäckande vadare: den relativa betydelsen av predation och trampskador av betesdjur. Ornis Svecica 15:89-96.

Ottvall, R. 2015. Åtgärdsprogram för hotade vadare på strandängar, 2015–2019. Swedish Environmental Protection Agency, Report 6680.

Pakanen, V. M., Thorup, O. 2016. Apparent adult survival in the critically endangered Baltic Dunlin Calidris alpina schinzii during a period of strong population decline. Bird Study 63: 293–302. http://dx.doi.org/10.1080/00063657.2016.1214812

Palmqvist, L. A. 1980. Bruket av våtängarna vid Nötmyran Västerfärnebo socken: ängsbruket och bebyggelsen i Hedbo by. Stockholm: Nordiska museet.

Pinchuk, P., N. Karlionova, Zhurauliou, D. 2005. Wader ringing at the Turov ornithological station, Pripyat Valley (S Belarus) in 1996-2003. The Ring 27:101.

Rydin, H., Borgegård, S.O. 1991. Plant characteristics over a century of primary sucession on islands: Lake Hjälmaren. Ecology 72(3):1089-1101.

Sjöbeck, M. 1973. Det sydsvenska landskapets historia och vård. AB Landstryck: Landskrona, Sweden.

Stanevičius, V., M. Mačiulis, S. Švažas. 2008. Breeding ecology of lapwing (Vanellus vanellus) in floodplains of the Nemunas River delta in 2006–2007. Ekologija 54:10-16.

Thorup, O. 1998. Ynglefuglene på Tipperne 1928-1992: bestandenes størrelse og ynglemuligheder i relation til skiftende driftsformer, prædation. fugtighedsforhold og vandmiljø [The breeding birds on Tipperne 1928-1992]. Dansk Ornitologisk Forening.

Thorup, O. 2003. Truede engfugle. Status for bestande og forvaltning i Danmark [Threatened meadowbirds. Status of populations and management in Denmark]. Dansk Ornitologisk Forening.

Thorup, O. 2016. Timing of breeding in Ruff Philomachus pugnax: a crucial parameter for management and use of wet grassland in Western Europe. Wader Study 123(1): 49-58.

Thorup, O., Preiksa, Z., Pehlak, H., Altemüller, M., Drews, H. 2011. Status of the Baltic Dunlin Calidris alpina in Lithuania. Wader Study Group Bull. 118(3):184–187.

Thorup, O. 1997. Langtidsstudier af Baltisk Ryle på Tipperne .[Long-term studies of Baltic dunlin at Tipperne] Dansk Orn. Foren. Tidsskrift 91:50-51.

Thorup, O. 2004. Suitable habitat management for Danish bird populations. In: Rannap, R., Briggs, L., Lotman, K., Lepik, I., Rannap, V. Coastal meadow management. Best practice guidelines. Ministry of the Environment of the Republic of Estonia, Tallinn, p. 44-51

# Appendix 2. Case study landscapes used for validation

## Kristianstad Vattenrike - past (Sweden)

The grassland landscape of Kristianstad (ca. 56°N, 14°E) was developed through traditional cultural grassland management along the lower part of Helge å River (Manton and Angelstam 2018). Christensen (1886) reported that about only a fifth of the Kristianstad grasslands were under plough during the medieval times. The two pre-Medieval cities Åhus and Vä declined after Kristianstad was developed from 1614 as a centre for defence of this region as a part of Denmark. The inaccessible wetlands had a protective military function until the city was ceded to Sweden in 1658. The grasslands in this lowland area were a key resource that was actively managed by flooding to overcome deficiency of nutrients (Dünkelberg 1873, Zachrison 1922, Emanuelsson and Möller 1990, Wallin 2011). This provided a foundation of animal husbandry, which was particularly favourable in areas subject to flooding. To conclude, the area of wet grasslands were at least two orders of magnitude more common prior to agricultural intensification in the latter part of the 19^th^ century. Local bird surveys from the 1930s (Jönsson et al. 2021) and reports from the preceding decades show that wader bird assemblages were still intact. The declines were clearly related to agricultural intensification.

## Friesland - past (The Netherlands)

Friesland is a prime example of how Dutch dairy farmers during centuries created and maintained a suitable breeding habitat for focal wader birds like the black-tailed godwit (e.g., Van Eerden et al. 2010). From the 16th century grasslands expanded through drainage programs and construction of dykes and polders (Hoeksema 2007). However, agricultural intensification has negatively affected the quality of meadow bird habitats. For example, southwest Friesland consists of 80% of uniform, intensively managed landscape with herb-poor meadows and low groundwater levels (ca. 53°N, 5°E). Groen et al. (2012) defined habitat for the focal wader species black-tailed godwit by groundwater level, vegetation typology, relief in the landscape, the occurrence of foot drains, land use and soil characteristics such as textures and peat to describe the landscape. To conserve local populations of black-tailed godwit, the vegetation should be herb-rich, contain foot drains and groundwater table should be high. Feeling torn between past food production demands as opposed to a shift to nature oriented goals, and lacking trust for government, farmers need a supportive structure to engage in nature conservation or restoration.

# References

Christensen, C. 1886. Agrarhistoriske Studier, Volume 2. Schubothe: Copenhagen, Denmark.

Dünkelberg, D. F. 1873. Handbok i ängsvattning – jemte ängars skötsel, vård och underhåll i allmänhet. Stockholm.

Emanuelsson, U., Möller, J. 1990. Flooding in Scania: A method to overcome the deficiency of nutrients in agriculture during the Nineteenth Century. The Agricultural History Review 38:127-148.

Hoeksema, R.J. 2007. Three stages in the history of land reclamation in the Netherlands. Irrigation and Drainage: The journal of the International Commission on Irrigation and Drainage 56(S1):113-126.

Groen, N.M., Kentie, R., de Goeij, P., Verheijen, B., Hooijmeijer, J.C., Piersma, T., 2012. A modern landscape ecology of Black-tailed Godwits: habitat selection in southwest Friesland, The Netherlands. Ardea 100(1):19-29.

Jönsson, M., Lithner, S., Svensson, S. 2021. Fågelfaunan vid Kävlingeån 1934-40. Anser (2):7-21.

Manton, M., Angelstam, P. 2018. Defining benchmarks for restoration of green infrastructure: A case study combining the historical range of variability of habitat and species’ requirements. Sustainability 10:326. doi:10.3390/su10020326. <http://www.mdpi.com/2071-1050/10/2>

Van Eerden, M. R., Lenselink, G., Zijlstra, M. 2010. Long-term changes in wetland area and composition in The Netherlands affecting the carrying capacity for wintering waterbirds. Ardea 98(3):265-282.

Wallin, N. 2011. ”Vatten gifver gräs” – Ängsvattning i Sverige och i synnerhet Malmöhus län. Examensarbete Högskolan i Kristianstad.

Zachrison, A. 1922. Nyodling, torrläggning och bevattning i Skåne 1800-1914. Lund.
